# Carcass Yield and Morphometric Characteristics of Semi-Intensive Pond-Cultured *Piaractus brachypomus* (Paco) at Three Commercial Weight Ranges in the Central Jungle of Peru

**DOI:** 10.3390/ani16152335

**Published:** 2026-07-31

**Authors:** Lizbeth Melendez-Atao, Luis Bazan-Alonso, Ide Unchupaico-Payano, Fernando Arauco-Villar, Noemi Mayorga-Sanchez

**Affiliations:** 1 Tropical Animal Science Program, Faculty of Agricultural Sciences, Universidad Nacional del Centro del Perú, Satipo 12261, Peru; e_2024280110f@uncp.edu.pe; 2 Faculty of Animal Science, Universidad Nacional del Centro del Perú, Huancayo 12000, Peru; iunchupaico@uncp.edu.pe (I.U.-P.); farauco@uncp.edu.pe (F.A.-V.); nmayorga@uncp.edu.pe (N.M.-S.)

**Keywords:** *Piaractus brachypomus*, aquaculture, allometry, morphometry, edible tissue recovery

## Abstract

The expansion of tropical aquaculture in the Peruvian Amazon has increased the need for quantitative information on processing performance and morphometric variation in commercially important native fish species. This study evaluated carcass yield, morphometric characteristics, intestinal traits, and edible tissue recovery of *Piaractus brachypomus* across three commercial weight categories (251–350 g, 351–450 g, and 451–550 g) in the central jungle of Peru. A total of 75 specimens were collected from a semi-intensive earthen-pond aquaculture unit at the Satipo station of the Tropical Animal Science Program, Universidad Nacional del Centro del Perú, located in Río Negro District, Satipo Province, Junín region, and analyzed under a completely randomized design with 25 fish per category. External morphometric measurements, intestinal variables, and processing yield indicators were recorded and analyzed using analysis of variance, regression models, principal component analysis, and Pearson correlation analysis. Most morphometric variables increased significantly with body weight, whereas tail length remained stable among categories. Principal component analysis explained 80.8% of total morphometric variation and showed clear separation among commercial weight groups. Intestinal length and relative intestinal ratios also increased progressively with fish size. Carcass yield exhibited a moderate decrease with increasing body weight, concomitant with a substantial increase in edible tissue recovery in larger fish. Fresh body weight showed a strong positive relationship with edible tissue recovery and emerged as the main predictor in multiple regression models. These results indicate that commercial weight influences body conformation, digestive development, and processing performance in *P. brachypomus*, providing information that may support harvest optimization and processing management in tropical aquaculture systems.

## Simple Summary

*Piaractus brachypomus*, locally known as paco, is one of the most important native fish species cultivated in the Peruvian Amazon. However, limited information is available regarding how harvest weight affects body shape, digestive development, and edible meat recovery under commercial farming conditions. This study evaluated fish from three commercial weight ranges commonly marketed in the central jungle of Peru. Measurements included body dimensions, intestinal characteristics, carcass yield, and edible tissue recovery. Most morphometric traits increased progressively with body weight, indicating coordinated body growth during fish development. Larger fish showed longer intestines and higher edible meat recovery, whereas smaller fish presented higher eviscerated carcass yield. Statistical analyses also revealed clear morphological differences among the three commercial categories. Fresh body weight was the main factor associated with edible tissue recovery, suggesting that harvest size can influence processing performance and product utilization. These findings provide useful information for improving harvest management, processing decisions, and production planning in paco aquaculture systems of the Peruvian Amazon.

## 1. Introduction

*Piaractus brachypomus*, commonly known as paco in Peru, pirapitinga in Brazil, and cachama blanca in Colombia and Venezuela, is a Neotropical characid fish native to the Orinoco and Amazon River basins [1,2]. This species is among the commercially important freshwater fish in South American aquaculture, owing to its rapid growth, omnivorous feeding habits with herbivorous tendency, adaptability to intensive culture systems, and high market acceptance [3,4]. In Peru, *P. brachypomus* aquaculture has expanded significantly in the central jungle region (Selva Central), particularly in Junín, where favorable climatic conditions and abundant water resources support year-round production [5].

The Peruvian aquaculture sector has experienced substantial growth over the past decade, with native Amazonian species contributing to food security and rural economic development [6]. According to recent FAO statistics, global aquaculture production continues to expand, with freshwater fish representing approximately 62% of total aquaculture output [7]. Within this context, *P. brachypomus* is increasingly cultivated by small- and medium-scale farmers in tropical regions, contributing to both domestic consumption and regional markets [8]. Production manuals and institutional reports from Peruvian Amazonian aquaculture systems have noted variable processing performance in *P. brachypomus* across harvest sizes, supporting the relevance of the weight ranges evaluated in the present study.

Despite the commercial importance of *P. brachypomus*, information remains limited concerning the relationship between harvest weight and processing characteristics. Carcass yield—defined as the proportion of edible or marketable product obtained after processing—is a fundamental parameter for economic viability in aquaculture operations [9,10]. Previous studies on related Serrasalmidae species, including *Colossoma macropomum* (tambaqui) and interspecific hybrids, have demonstrated that body weight significantly influences morphometric ratios, fillet yield, and overall processing efficiency [3,11,12]. However, comprehensive data specifically addressing *P. brachypomus* at commercially relevant weight ranges in Peruvian production systems remain limited.

Morphometric characterization provides essential information for understanding growth patterns, optimizing feeding strategies, and establishing quality standards for market differentiation [13,14]. External morphometric measurements, including standard length, body proportions, and fin dimensions, serve as indicators of fish condition, growth performance, and genetic potential [15]. Additionally, internal characteristics such as intestinal length and relative intestinal length ratio offer insights into digestive physiology and feeding ecology, which are particularly relevant for herbivorous and omnivorous species [16,17].

Recent research has examined weight-specific processing strategies for improving economic returns from aquaculture production [3,18]. Studies on *P. brachypomus* and related species have shown that carcass composition, fillet yield, and meat quality vary significantly with body size, suggesting that harvest weight selection should be optimized based on target market requirements [19,20]. Furthermore, understanding the allometric relationships between body weight and morphometric traits enables producers to predict processing outcomes and adjust production cycles accordingly [21].

In the Satipo province of Junín region, *P. brachypomus* is typically marketed at weights ranging from 250 to 550 g, representing the most common commercial size classes in local and regional markets [22]. However, systematic evaluation of how these weight categories differ in terms of carcass yield, morphometric characteristics, and processing efficiency has not been previously documented for this production area. Such information supports evidence-based recommendations for optimal harvest weight, improving value chain efficiency, and enhancing market competitiveness.

The objective of this study was to determine and compare carcass yield and morphometric characteristics of *P. brachypomus* across three commercial weight ranges (251–350 g, 351–450 g, and 451–550 g) in the central jungle of Peru. Specific aims included: (1) quantifying differences in raw carcass yield, fried carcass yield, and edible portion percentages among weight categories; (2) characterizing external morphometric measurements and their allometric relationships with body weight; (3) evaluating intestinal characteristics as indicators of feeding habit and digestive capacity; and (4) providing practical recommendations for harvest weight optimization based on processing efficiency and market requirements. This research provides standardized characterization data to support the development of *P. brachypomus* aquaculture in the Peruvian Amazon.

## 2. Materials and Methods

### 2.1. Study Area and Sample Collection

The study was conducted in the districts of Satipo and Río Negro, Satipo province, Junín region, Peru, located in the central jungle (Selva Central) of the Peruvian Amazon. The study area is situated between 11°15′ S latitude and 74°38′ W longitude, at an average elevation of 650 m above sea level. The region is characterized by a tropical humid climate with a mean annual temperature of 24.5 °C, relative humidity of 85%, and annual precipitation ranging from 1800 to 2500 mm [23].

Fish specimens were obtained from a single semi-intensive earthen-pond aquaculture unit at the Satipo station of the Tropical Animal Science Program, Universidad Nacional del Centro del Perú, located in Río Negro District, Satipo Province, Junín region. All fish were reared under the same husbandry conditions, including feeding with commercial extruded pellets (28% crude protein), natural pond productivity supplementation, and standard water quality management practices. Fish were harvested at commercial size after approximately 8–10 months of grow-out culture, depending on initial stocking size and growth performance.

**Figure 1:**
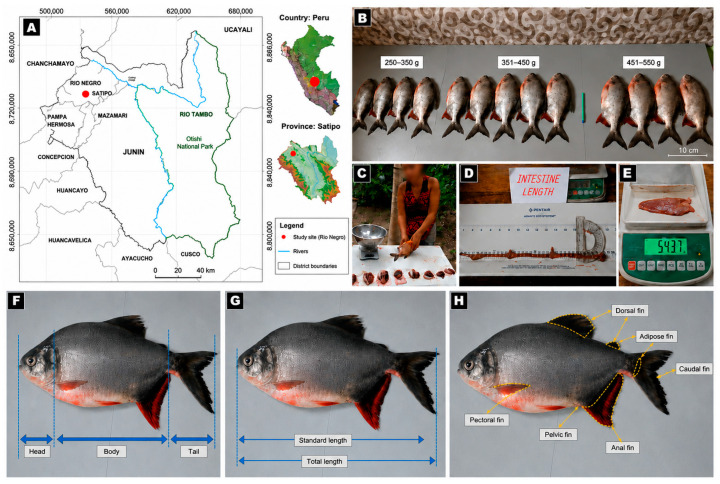
Study area, commercial weight categories, processing procedures, and morphometric measurements evaluated in *Piaractus brachypomus* from the central jungle of Peru. (**A**) Geographic location of the study area in Río Negro District, Satipo Province, Junín Region, Peru, including regional and provincial reference maps and sampling site location. (**B**) Commercial weight categories evaluated in the study: 251–350 g, 351–450 g, and 451–550 g. (**C**) Evisceration and carcass-processing procedure performed prior to yield analysis. (**D**) Measurement of intestinal length after dissection and straightening of the digestive tract. (**E**) Determination of edible portion weight using a digital balance. (**F**) External body regions considered for morphometric characterization, including head, trunk, and tail regions. (**G**) Standard length and total length measurements used for morphometric analysis. (**H**) External fin measurements and anatomical structures evaluated, including dorsal, adipose, caudal, anal, pelvic, and pectoral fins.

A total of 75 *P. brachypomus* specimens were randomly selected and classified into three commercial weight categories: Category 1 (C1): 251–350 g; Category 2 (C2): 351–450 g; and Category 3 (C3): 451–550 g. These weight ranges represent the most common commercial size classes marketed in the region. Fish were transported alive in oxygenated containers to the processing facility at the Universidad Nacional del Centro del Perú, Satipo campus, where all measurements and processing procedures were conducted within 24 h of harvest. Sex was not determined prior to grouping, as reliable external sexual differentiation in *P. brachypomus* at these body sizes (250–550 g) is not feasible without histological examination of gonadal tissue. Because all specimens came from a single production unit under standardized management, farm-of-origin variability was not considered a confounding factor in the present design. An overview of the study area, commercial weight categories, processing procedures, and morphometric measurements evaluated in this study is presented in Figure 1.

### 2.2. Experimental Design

The experiment followed a completely randomized design (CRD) with three treatments corresponding to the three commercial weight categories, with 25 replicates (individual fish) per treatment. The independent variable was commercial weight category (C1, C2, C3), and dependent variables included fresh weight, 11 external morphometric measurements, intestinal characteristics, and four carcass yield indicators. Prior to measurements, fish were humanely euthanized using hypothermic shock (immersion in ice-water slurry at 0–2 °C for 10 min) following ethical guidelines for fish handling in research [24]. All experimental procedures were conducted in accordance with institutional animal care protocols.

### 2.3. Morphometric Measurements

External morphometric measurements were obtained using a digital caliper (precision ± 0.01 mm) and an ichthyometer with a millimetric scale. Fresh body weight (*FW*) was measured using a digital balance with precision ± 0.01 g. All measurements were performed following standardized ichthyometric procedures adapted from Strauss and Bond [25]. To improve measurement consistency, all morphometric evaluations were conducted by the same trained operator under standardized laboratory conditions. Digestive morphometric traits were measured immediately after dissection. The digestive tract was carefully removed, straightened without excessive stretching, and measured using a flexible measuring tape. Morphometric and digestive variables evaluated in the present study are summarized in Table 1.

This standardized morphometric framework was used to evaluate ontogenetic variation in body conformation, digestive development, and processing-related traits across commercial weight categories.

### 2.4. Yield and Processing Calculations

Processing yield evaluation was conducted using a standardized protocol designed to simulate commercial fish-processing conditions. After euthanasia and morphometric evaluation, each specimen was individually processed through sequential stages including evisceration, frying, and edible tissue separation. All weights were recorded using a digital balance with precision ± 0.01 g immediately after each processing stage to minimize measurement bias associated with moisture loss. The processing protocol applied constitutes primary processing, encompassing evisceration (removal of viscera, gills, and scales) followed by thermal treatment (deep-frying) and manual edible tissue separation; no secondary processing steps such as freezing, canning, or filleting for frozen storage were performed.

Subsequently, carcasses were fried under a standardized artisanal processing simulation designed to reproduce the common local preparation of whole fried paco in the central jungle of Peru. Each eviscerated carcass was fried individually in vegetable oil at approximately 180 °C for 8 min, using the same frying container, heat source, operator, and handling procedure for all specimens. The frying step was not intended to represent an industrial thermal-processing protocol, but rather a controlled approximation of the practical local-market preparation of “paco frito”. Fried carcass weight (*FWc*) was recorded immediately after frying and draining.

(1) $$CY\left(\%\right)=\frac{EW}{FW}\times 100$$

where *CY* is carcass yield (%), *EW* is eviscerated weight (g), and *FW* is fresh body weight (g).

(2) $$FCY\left(\%\right)=\frac{{FW}_{c}}{FW}\times 100$$

where *FCY* is fried carcass yield (%), *FW_c_* is fried carcass weight (g), and *FW* is fresh body weight (g).

(3) $$EPT\left(\%\right)=\frac{EP}{FW}\times 100$$

where *EPT* is edible part relative to total weight (%), *EP* is edible portion weight (g), and *FW* is fresh body weight (g).

(4) $$EPC\left(\%\right)=\frac{EP}{EW}\times 100$$

where *EPC* is edible part relative to carcass weight (%), *EP* is edible portion weight (g), and *EW* is eviscerated carcass weight (g). All processing procedures were performed by the same trained personnel to reduce operator-dependent variability and ensure methodological consistency among specimens and commercial categories. In the Peruvian central jungle, *P. brachypomus* is predominantly commercialized as whole fried fish (“paco frito”), which is the practical justification for using *FCY* as a processing indicator for this regional market. Because *FCY* incorporates moisture loss and oil absorption during frying, *FCY* values are not directly comparable with fillet-yield values reported in non-fried processing studies.

### 2.5. Allometric and Multivariate Analyses

To evaluate growth patterns and morphological variation among commercial weight categories, both allometric and multivariate statistical approaches were applied. Length–weight and intestinal allometric relationships were analyzed using log_10_-transformed linear regression models according to the general allometric equation:

(5) $$\log\left(Y\right)=\log\left(a\right)+b\;log(X)$$

where *Y* represents the dependent biological variable, *X* the independent variable, *a* the intercept, and *b* the allometric coefficient. Values of *b* < 1 were interpreted as negative allometry, *b* = 1 as isometric growth, and *b* > 1 as positive allometry. This classification criterion follows the standard framework established by Huxley (1932) for the interpretation of allometric growth coefficients. Linear regressions were used to evaluate the relationships between total length and fresh weight, intestinal length and fresh weight, and intestinal length and standard length. Coefficients of determination (*R*^2^), regression slopes, and significance levels were calculated for all models. 95% confidence intervals for each allometric coefficient (*b*) were additionally calculated to support the classification of allometric type.

Principal component analysis (PCA) was performed using standardized morphometric variables to identify the main axes of morphological variation and evaluate clustering patterns among commercial categories. PCA was applied specifically to: (1) quantify the proportion of total morphometric variation attributable to a general body-size axis versus secondary independent axes; (2) evaluate whether the commercial weight categories correspond to biologically differentiated morphological groups rather than arbitrary market divisions; and (3) identify variables, such as tail length, whose growth pattern is independent of overall body size. Prior to analysis, variables were centred and scaled to unit variance to minimize the influence of measurement magnitude. Eigenvalues, percentage variance explained, and variable loadings were extracted for interpretation of principal components. PCA ordination plots were generated using the first two principal components.

Pearson correlation analysis was conducted to evaluate associations among morphometric, digestive, and processing-yield variables. Correlation coefficients were visualized through a heatmap based on the Pearson correlation matrix, allowing identification of positive and negative associations among biological and processing traits. Additionally, multiple linear regression models were fitted to identify the best predictors of carcass yield (*CY*) and edible part relative to total weight (*EPT*). Predictor variables were incorporated sequentially according to their biological relevance, including fresh weight, trunk length, intestinal length, and standard length. The hierarchical entry order applied was: (1) *FW* first, as the primary body-condition indicator with the strongest a priori biological association with the yield variables; (2) *TrL* second, as the body region containing the greatest commercial muscle mass; (3) *IL* third, as a proxy for visceral development relevant to *CY*; and (4) *SL* fourth, as an alternative whole-body size metric. Model performance was evaluated using coefficients of determination (R^2^ and adjusted R^2^), analysis of variance (F-test), and Akaike Information Criterion (AIC). Lower AIC values were interpreted as indicating better model fit with reduced complexity. Variance Inflation Factors (VIFs) were additionally calculated for all multi-predictor models to assess multicollinearity among these correlated morphometric variables.

### 2.6. Statistical Analysis

Data were organized in Microsoft Excel 2019 (Microsoft Corporation, Redmond, WA, USA) and analysed using R statistical software version 4.3.1 (R Core Team, Vienna, Austria). Descriptive statistics were expressed as mean ± standard deviation for all evaluated variables within each commercial weight category. Prior to inferential analyses, normality of data distribution was assessed using the Shapiro–Wilk test, while homogeneity of variances was evaluated using Levene’s test. Based on the present analysis of the raw dataset, most but not all variables satisfied the assumptions of parametric analysis: 12 of 17 evaluated variables (*FW*, *TL*, *SL*, *TrL*, *TaL*, *DFL*, *PFL*, *PvFL*, *AFL*, *CFL*, *FCY*, *EPT*) showed deviations from normality (Shapiro-Wilk *p* < 0.05), and 7 of 17 variables (*TrL*, *DFL*, *PvFL*, *GR*, *RIL*, *FCY*, *EPT*) showed heterogeneity of variances (Levene’s *p* < 0.05); full results are reported in Supplementary Table S1. Given these deviations, one-way analysis of variance (ANOVA) was retained as the primary inferential test, and was additionally cross-validated against its non-parametric equivalent, the Kruskal–Wallis test, for every evaluated variable. When significant differences were detected (*p* < 0.05), Duncan’s multiple range test was applied for post hoc comparisons to identify homogeneous statistical groups among treatments. Duncan’s test was selected because it is widely used and recommended for agricultural and aquaculture experiments with balanced designs. As a robustness check, both Tukey’s HSD test (parametric) and the Games–Howell test (non-parametric-robust, not assuming equal variances or normality) were additionally applied to all 17 variables. Statistical significance was established at α = 0.05. Results in tables are presented using superscript lowercase letters to indicate significant differences according to Duncan’s test. Complete ANOVA and Kruskal–Wallis statistics are reported in Supplementary Table S2; Tukey HSD and Games–Howell pairwise results are reported in Supplementary Tables S3 and S4, respectively. For all 17 variables, Kruskal–Wallis reached the same conclusion regarding statistical significance as ANOVA, and Games–Howell identified the same number of significant pairwise comparisons as Tukey HSD.

## 3. Results

### 3.1. Morphometric Growth Patterns Across Commercial Weight Categories

Most external morphometric measurements increased significantly across the three commercial weight categories (Table 2). Total length increased from 23.82 ± 0.52 cm in C1 to 27.31 ± 0.83 cm in C3, while standard length ranged from 19.08 ± 0.83 cm to 22.21 ± 0.68 cm. Trunk length exhibited the most pronounced relative increase, rising from 13.82 ± 0.43 cm in C1 to 18.90 ± 1.36 cm in C3, representing a 36.8% increase. In contrast, tail length showed no significant variation across categories (*p* = 0.754; F(2,72) = 0.283), indicating developmental stability in this morphometric trait. Fin measurements, including dorsal, pectoral, pelvic, anal, and caudal fins, increased significantly (*p* < 0.01) with body weight, reflecting coordinated ontogenetic scaling of locomotor and stabilizing structures.

**Figure 2:**
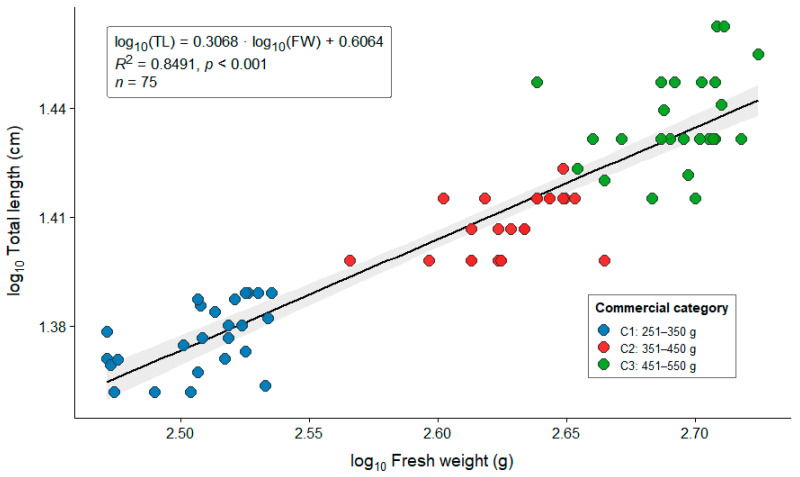
Length–weight relationship (log_10_-transformed) of *Piaractus brachypomus* across three commercial weight categories (C1: blue, C2: red, C3: green). Solid line: ordinary least-squares regression; shaded band: 95% confidence interval. Regression equation and coefficient of determination are shown in the upper-left inset.

Log-transformed regression analysis showed a strong and significant relationship between fresh weight and total length across the 75 specimens (Figure 2): log10(*TL*) = 0.3068 × log10(*FW*) + 0.6064 (R^2^ = 0.8491, *p* < 0.001), 95% CI for b: 0.2766–0.3369. This result indicates that total length increased predictably with body weight, although the slope below 1 suggests that length increased at a proportionally lower rate than body mass. Therefore, heavier fish tended to show relatively greater body mass accumulation per unit of length, consistent with increasing body robustness across commercial weight categories.

**Figure 3:**
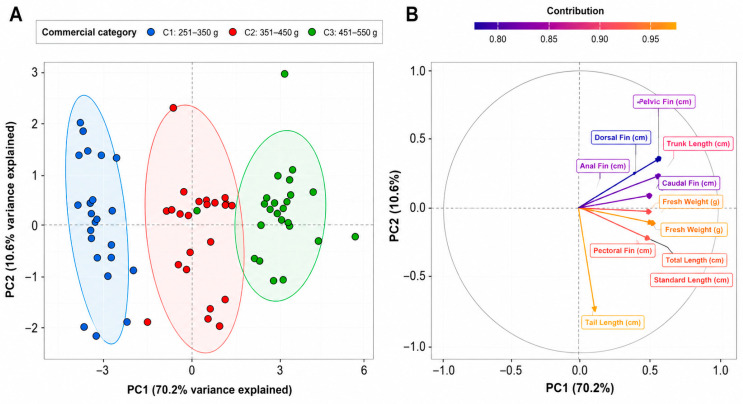
Principal component analysis (PCA) of ten external morphometric variables of *Piaractus brachypomus* (*n* = 75). (**A**) PCA score plot showing the distribution of specimens according to commercial weight categories (C1: 251–350 g, blue; C2: 351–450 g, red; C3: 451–550 g, green). Ellipses represent the dispersion of each category. (**B**) PCA loading plot showing the contribution of the morphometric variables to the first two principal components. Arrows indicate variable loadings, and the percentage of variance explained is shown on each axis.

Principal component analysis of the ten external morphometric variables (Figure 3) produced two principal components explaining 70.2% and 10.6% of the total variance, respectively, accounting for a cumulative 80.8% of morphometric variation. PC1 was positively associated with overall body growth and size-related traits, showing high scaled loadings for fresh weight (*FW* = 0.974), total length (*TL* = 0.949), standard length (*SL* = 0.921), trunk length (*TrL* = 0.897), pectoral fin length (*PFL* = 0.904), pelvic fin length (*PvFL* = 0.859), anal fin length (*AFL* = 0.859), and caudal fin length (*CFL* = 0.860). In contrast, PC2 was dominated primarily by tail length (*TaL* = 0.984), indicating that this variable represented a secondary axis of morphological variation that was weakly associated with the general body-size gradient. Complete PC1 and PC2 loading values are provided in Supplementary Table S5.

The ordination plot revealed a clear separation among the three commercial weight categories along PC1, with C1 positioned at negative scores and C3 concentrated at positive scores, reflecting progressive morphometric differentiation with increasing body weight. Minimal overlap among clusters supports the existence of biologically distinct growth stages across commercial classes. Furthermore, the tighter dispersion observed in C2 and C3 indicates reduced morphometric variability at larger body sizes, which may have practical implications for harvest standardization and processing uniformity in commercial production systems.

**Figure 4:**
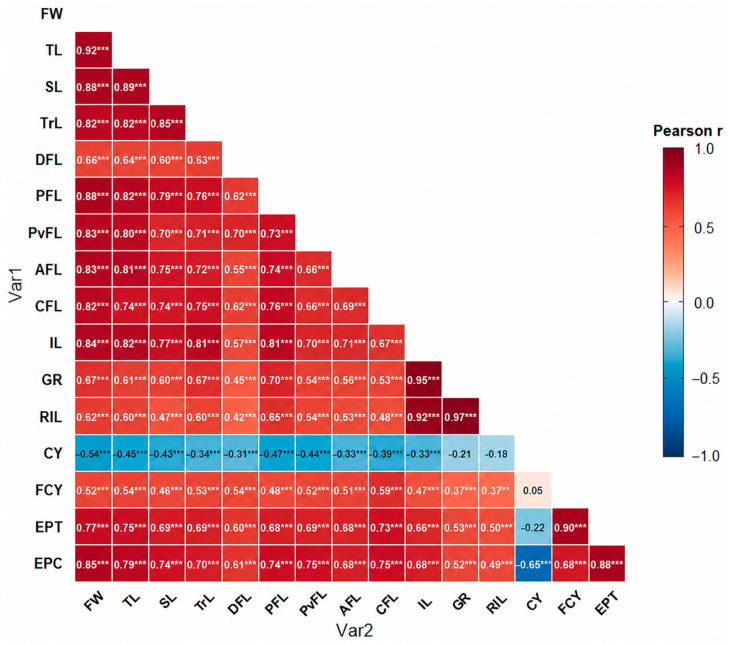
Pearson correlation heatmap for all morphometric and yield variables of *Piaractus brachypomus* (*n* = 75). Color scale ranges from dark blue (*r* = −1) to dark red (*r* = +1). Numerical values are shown within each cell. Abbreviations: *FW* = fresh weight; *TL* = total length; *SL* = standard length; *TrL* = trunk length; *DFL*–*CFL* = fin lengths; *IL* = intestine length; *GR* = *IL*/*TL* ratio; *RIL* = *IL*/*SL* ratio; *CY* = carcass yield; *FCY* = fried carcass yield; *EPT* = edible part/total weight; *EPC* = edible part/carcass weight. Significance levels: ** *p* < 0.01; *** *p* < 0.001.

Pearson correlation analysis (Figure 4) revealed strong positive intercorrelations among most external morphometric variables, indicating coordinated somatic growth across commercial weight categories. Fresh weight was highly correlated with total length (r = 0.92), standard length (r = 0.88), trunk length (r = 0.82), and intestine length (r = 0.84), confirming the integrated scaling of external and visceral growth traits. Similarly, fin measurements exhibited consistently positive associations with body size variables (r = 0.55–0.88), reflecting proportional appendicular development during ontogeny.

Digestive traits showed particularly strong internal associations, with intestine length being highly correlated with the general intestinal ratio (*GR*; r = 0.95) and relative intestine length (*RIL*; r = 0.92). In contrast, carcass yield (*CY*) displayed moderate negative correlations with most morphometric variables, especially fresh weight (r = −0.54) and edible part/carcass weight (*EPC*; r = −0.65), suggesting that larger fish allocate proportionally less mass to the eviscerated carcass fraction. Conversely, edible part indicators showed strong positive correlations with fresh weight and morphometric dimensions, particularly between *EPT* and *EPC* (r = 0.88), highlighting the close relationship between somatic growth and edible tissue recovery.

### 3.2. Intestinal Allometry and Digestive Traits

Intestinal morphometric traits increased significantly across weight categories (Table 3). Absolute intestinal length (*IL*) rose from 38.83 ± 5.04 cm (C1) to 45.08 ± 3.30 cm (C2) and 54.80 ± 3.24 cm (C3), representing a 41.1% total increase. The *IL*/*TL* ratio increased from 1.63 ± 0.22 to 2.01 ± 0.12, and the relative intestinal length (*IL*/*SL*) from 2.04 ± 0.29 to 2.47 ± 0.16. All pairwise comparisons were significant (Duncan test, *p* < 0.01), indicating progressive digestive system development across commercial weight ranges.

**Figure 5:**
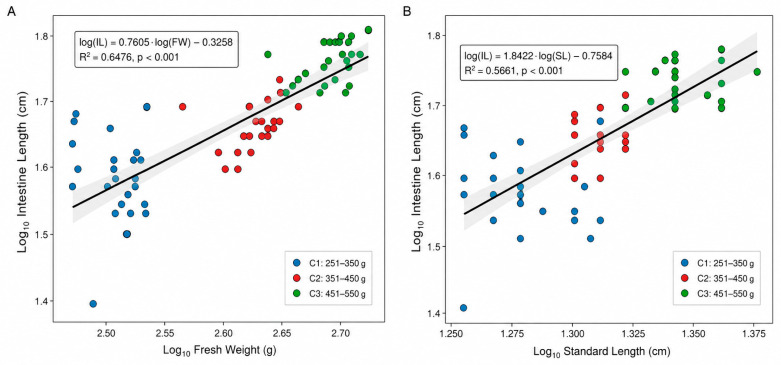
Allometric regression of intestinal length (*IL*) on fresh body weight (**A**) and standard length (**B**) for *Piaractus brachypomus* (*n* = 75), plotted on log_10_-transformed axes. Solid lines: ordinary least-squares regression; shaded bands: 95% confidence intervals. Regression equations, coefficients of determination (R^2^), and significance levels are shown in upper-left insets. Points are coloured by weight category.

Allometric regression of intestinal length on body weight (Figure 5A) yielded: log(*IL*) = 0.7605 · log(*FW*) + (−0.3002) (R^2^ = 0.648, *p* < 0.001). The scaling exponent b = 0.761 (95% CI: 0.6296–0.8913) < 1.0 indicates negative allometry—intestinal length grows at a slower rate than body weight, meaning that as fish become heavier, the gut-to-body-weight ratio decreases slightly. Regression against standard length (Figure 5B) yielded: log(*IL*) = 1.8422 · log(*SL*) + (−1.3418) (R^2^ = 0.566, *p* < 0.001), with b = 1.842 (95% CI: 1.4660–2.2183) > 1 indicating positive allometry relative to body length—the intestine elongates disproportionately as standard length increases. The *IL*/*SL* values exceeding 2.0 across all categories are characteristic of omnivore–herbivore species with high plant material consumption [3,4], consistent with the natural diet of *P. brachypomus* in Amazonian floodplain environments. The reported confidence intervals confirm that both allometric coefficients differ significantly from isometry (b = 1), supporting the negative and positive allometry classifications, respectively, following the criterion described by Huxley [26].

### 3.3. Processing Yield and Edible Recovery

Processing yield indicators varied significantly among weight categories for three of four variables (Table 4). Carcass yield (*CY*) declined from 87.09 ± 6.38% (C1) to 79.72 ± 6.79% (C3), F(2,72) = 9.68 (*p* = 0.0002), reflecting the proportionally greater visceral mass in larger fish. In contrast, fried carcass yield (*FCY*) increased from 59.79 ± 3.83% (C1) to 66.10 ± 3.11% (C3), indicating improved water-retention capacity and muscle density at higher body weights. The edible part as a proportion of total weight (*EPT*) showed the largest increase among the evaluated processing indicators, rising from 37.05 ± 3.32% (C1) to 47.28 ± 2.17% (C2) and 49.54 ± 3.24% (C3)—a 33.7% absolute gain.

**Figure 6:**
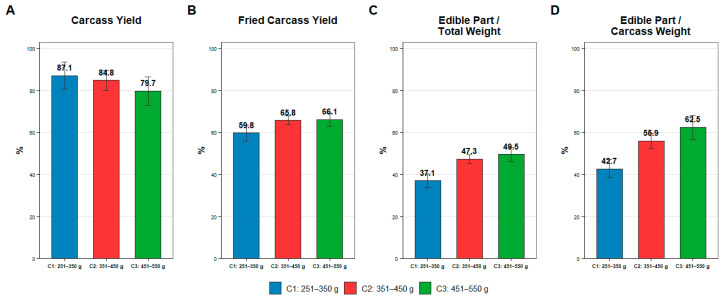
Processing yield indicators (mean ± SD) of *Piaractus brachypomus* across three commercial weight categories (C1: blue, C2: red, C3: green). Panels show (**A**) carcass yield (CY); (**B**) fried carcass yield (FCY); (**C**) edible part/total weight (EPT); and (**D**) edible part/carcass weight (*EPC*). Error bars represent ± 1 SD. Mean values are labelled above each bar.

Visual comparison of yield indicators across categories (Figure 6) highlights the divergent trajectories of carcass yield and edible recovery. While *CY* decreases monotonically with body weight, *EPT* and *EPC* increase substantially, with the largest gains occurring between C1 and C2.

**Figure 7:**
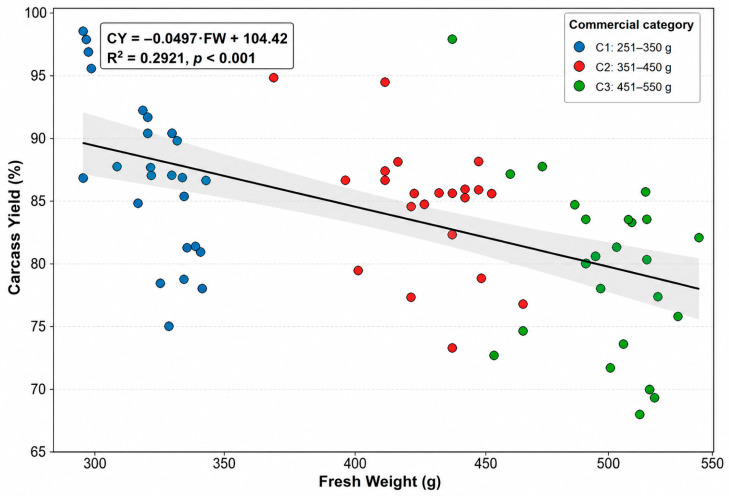
Relationship between fresh body weight (g) and carcass yield (%) in *Piaractus brachypomus* (*n* = 75). Points are coloured by weight category (C1: blue, C2: red, C3: green). Solid line: linear regression; shaded band: 95% confidence interval. Regression equation and R^2^ are shown in the upper-left inset.

The relationship between fresh weight and carcass yield (Figure 7) was negative and significant (*CY* = −0.0497 · *FW* + 103.66; R^2^ = 0.292, *p* < 0.001), confirming that heavier fish yield a lower proportion of eviscerated carcass. However, the modest R^2^ value (29.2%) suggests substantial individual variation in visceral fat deposition and organ development that is not fully captured by body weight alone.

**Figure 8:**
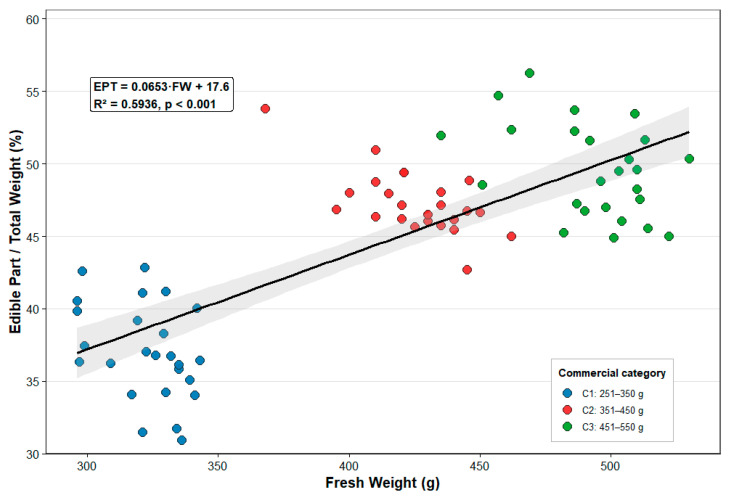
Relationship between fresh body weight (g) and edible part/total weight (%) in *Piaractus brachypomus* (*n* = 75). Points are coloured by weight category (C1: blue, C2: red, C3: green). Solid line: linear regression; shaded band: 95% confidence interval. Regression equation and R^2^ are shown in the upper-left inset.

A supplementary continuous regression analysis across the full individual weight range, including a comparison between linear and second-degree polynomial fits, is provided in Supplementary Figure S1. In contrast, the relationship between fresh weight and edible part recovery (*EPT*) was strongly positive (*EPT* = 0.0653 · *FW* + 16.23; R^2^ = 0.594, *p* < 0.001; Figure 8), indicating that fresh weight is an important predictor of edible tissue recovery under the evaluated processing conditions. The slope indicates that each additional 100 g of body weight corresponds to approximately 6.5 percentage points of additional edible meat recovery. The 95% confidence bands narrow at intermediate weights (380–450 g), suggesting reduced prediction uncertainty in the C2 range and supporting C2–C3 as relevant harvest ranges from a processing-yield perspective. A supplementary polynomial (second-degree) fit of *EPT* on *FW* across the full individual weight range showed a statistically significant quadratic term (R^2^ = 0.643 vs. 0.594 for the linear model; see Supplementary Figure S1), indicating that the rate of increase in *EPT* may moderate somewhat at the highest body weights (approximately 480–530 g) rather than remaining strictly linear across the entire range.

### 3.4. Predictors of Processing Efficiency

Multiple linear regression models were fitted to identify the best morphometric predictors of carcass yield and edible recovery (Table 5). For carcass yield, fresh weight alone explained 29.2% of the variance (Adj. R^2^ = 0.282, F = 30.12, *p* < 0.0001). Adding trunk length improved the model marginally (Adj. R^2^ = 0.310), and further inclusion of intestinal length yielded Adj. R^2^ = 0.331. The addition of standard length in Model 4 provided no meaningful improvement (ΔAIC < 1), indicating that carcass yield is only moderately predictable from external measurements, likely due to the high individual variability in visceral fat content. Variance Inflation Factor (VIF) analysis of the multi-predictor models revealed very high multicollinearity among *FW*, *TrL*, *IL*, and *SL* (VIF ranging from approximately 104 in the two-predictor model to nearly 200 in the four-predictor model; full values in Table 5), which is consistent with the strong intercorrelations documented in the Pearson correlation matrix (Figure 4) and explains why individual predictor coefficients beyond *FW* are statistically unstable despite small gains in overall R^2^. For this reason, multi-predictor models were interpreted at the model-comparison level rather than through individual coefficients. Only the *FW*-only models were considered suitable for direct biological interpretation, whereas coefficients from models including *TrL*, *IL*, and *SL* were treated as unstable due to severe multicollinearity.

For edible recovery (*EPT*), fresh weight was by far the dominant predictor (R^2^ = 0.594, Adj. R^2^ = 0.588, F = 106.63, *p* < 0.0001). The addition of trunk length, intestinal length, and standard length in Models 2–4 provided negligible improvements (ΔAdj. R^2^ < 0.01 per variable), confirming that fresh weight is the primary driver of edible meat yield. This finding has direct practical implications: body weight at harvest is the most reliable and easily measurable indicator of processing efficiency in *P. brachypomus* aquaculture operations.

## 4. Discussion

### 4.1. Carcass Yield Dynamics Across Weight Categories

The progressive decline in carcass yield (*CY*) with increasing body weight observed in the present study indicates an ontogenetic shift in body component allocation in *Piaractus brachypomus.* Although larger fish exhibited lower eviscerated carcass percentages, edible tissue recovery increased substantially across commercial categories, suggesting that somatic growth in this species is accompanied by disproportionate accumulation of muscle tissue relative to skeletal and visceral fractions [27]. This interpretation is supported by the simultaneous increase in fried carcass yield (*FCY*), edible part relative to total weight (*EPT*), and edible part relative to carcass weight (*EPC*), particularly in the C3 category. This pattern—lower carcass yield yet higher edible recovery in larger fish—reflects the ontogenetic partitioning of somatic growth: visceral mass expands allometrically, depressing *CY*, while skeletal muscle deposition accelerates disproportionately, enhancing *EPT* and *EPC*.

The reduction in *CY* from 87.09% in C1 to 79.72% in C3 is consistent with observations reported for other Serrasalmidae species, where increasing body size is associated with greater visceral development and higher deposition of internal tissues removed during processing. Cirne et al. reported similar tendencies in *Colossoma macropomum*, indicating that heavier fish may allocate proportionally more biomass to viscera and non-commercial fractions [20]. Likewise, the marked increase in intestinal length documented in the present study supports the hypothesis that digestive tract expansion contributes substantially to the decline in eviscerated yield in larger specimens [28].

In contrast, the increase in *EPT* and *EPC* suggests that muscle deposition becomes progressively more efficient with increasing body size. Ribeiro et al. similarly reported positive associations between body weight and carcass recovery in *P. brachypomus*, suggesting that morphometric growth in this species favours commercially valuable tissue accumulation at advanced growth stages. From a production perspective, the 33.7% increase in edible portion between C1 and C3 represents a substantial improvement in processing efficiency that may compensate for the moderate reduction in raw carcass yield [21].

The regression analyses further reinforce this interpretation. The negative relationship between fresh weight and carcass yield indicates that larger fish progressively allocate more biomass to visceral structures, whereas the strong positive relationship between fresh weight and edible recovery demonstrates that harvest weight remains the main determinant of commercially usable tissue [29]. The moderate explanatory power of the *CY* model (R^2^ = 0.292) suggests that carcass yield is influenced not only by body size but also by individual variability in visceral development and tissue composition. Part of this unexplained variability may also reflect individual-level factors not captured in the present dataset, such as variation in visceral fat deposition unrelated to body size, and undetermined sex-related differences in gonadal and visceral mass. Since all specimens originated from a single production system with standardized husbandry management, farm-of-origin variability is not a plausible contributor to this residual variance. Conversely, the higher predictive capacity observed for *EPT* (R^2^ = 0.594) indicates that edible recovery is more tightly linked to somatic growth dynamics.

From a practical standpoint, these findings indicate that harvest strategies should depend on the intended market destination. Smaller fish may be more appropriate for whole-fish commercialization due to their higher eviscerated yield, whereas larger fish provide superior edible recovery and therefore greater suitability for fillet production and value-added processing [30]. The intermediate category (C2) appears particularly attractive because it combines relatively high carcass yield with markedly improved edible recovery, representing a biologically balanced harvest range from a processing-yield perspective. We note that this is a processing-yield-based observation; an economic optimization claim would additionally require feed cost, growth-cycle duration, and market price data, which were not collected in the present study.

### 4.2. Morphometric Allometry and Growth Patterns

The morphometric results indicated coordinated somatic growth across commercial categories, with most body dimensions increasing significantly with fresh weight. The high correlations among fresh weight, total length, standard length, trunk length, and fin measurements indicate integrated ontogenetic development, consistent with the coordinated growth patterns described for *P. brachypomus* and related Serrasalmidae species [31].

Among the evaluated traits, trunk length exhibited the greatest proportional increase, suggesting preferential expansion of the central body region during growth. Because this region contains most commercially valuable muscle mass, the observed positive allometry supports the increase in edible recovery observed in larger fish. Similar morphometric tendencies were reported by Parés-Casanova [14] and Ribeiro et al. [21], who identified strong associations between trunk-related measurements and carcass yield traits in *P. brachypomus*.

The log-transformed length–weight relationship revealed a strong association between total length and fresh weight (R^2^ = 0.849), indicating predictable somatic growth across commercial categories. However, the scaling coefficient suggested that body mass increased proportionally faster than body length, implying progressive body deepening and increased tissue deposition as fish approached larger commercial sizes. This pattern is biologically relevant because it reflects increasing muscular robustness rather than simple linear elongation, which directly contributes to improved edible tissue recovery [32].

Principal component analysis reinforced these findings by showing that PC1 primarily represented a general body-size axis, as evidenced by the high positive scaled loadings for fresh weight, total length, standard length, trunk length, and most fin dimensions. Therefore, the separation among commercial weight categories along PC1 should be interpreted mainly as size-related morphometric differentiation rather than as evidence of fully size-independent shape divergence. This indicates that the evaluated weight classes reflect progressive body-size development within commercially relevant harvest ranges, rather than merely arbitrary market groupings. Moreover, the reduced dispersion observed in C2 and C3 suggests increasing morphometric uniformity at larger body sizes, a characteristic that may favour industrial processing standardization [33]. It should be noted, however, that because PC1 is dominated by variables correlated with overall body size, PCA in the present analysis primarily captures size-related covariation.

Interestingly, tail length showed minimal contribution to the general body-size gradient represented by PC1 and remained statistically stable across categories. PC2 was dominated by tail length, indicating that this trait varied independently from the main somatic growth trajectory. Similar regional heterogeneity in growth has been reported in Characiformes, where locomotor structures may exhibit greater developmental stability due to biomechanical constraints associated with swimming performance [34,35]. This interpretation supports the descriptive and applied value of PCA in the present study, while avoiding overinterpretation of the ordination as evidence of independent morphological specialization.

### 4.3. Intestinal Characteristics and Feeding Ecology

The progressive increase in intestinal length and intestinal ratios across commercial categories reflects substantial ontogenetic development of the digestive system in *P. brachypomus.* Relative intestinal length values exceeding 2.0 in all categories are characteristic of omnivorous–herbivorous fishes and support previous ecological descriptions of the species as predominantly herbivorous with opportunistic omnivory.

The positive relationship between intestinal dimensions and body size suggests increasing digestive capacity during growth. However, the allometric regressions revealed contrasting scaling patterns depending on the reference variable. Intestinal length increased more slowly than fresh weight (negative allometry), indicating that gut mass does not expand proportionally with total biomass accumulation. In contrast, intestine length increased disproportionately relative to standard length, suggesting progressive elongation of the digestive tract as body shape changes during ontogeny [16]. These results indicate that digestive development in *P. brachypomus* is not merely a consequence of increasing body size but represents an adaptive reorganization associated with feeding efficiency and nutrient assimilation. We note, however, that conclusions regarding feeding ecology and digestive efficiency drawn solely from intestinal length measurements are inherently limited; definitive characterization of dietary preference or digestive performance would require gastric content analysis, enzymatic activity assays, or stable isotope tracing, none of which were performed in the present study. The intestinal allometry results presented here should therefore be interpreted as anatomically consistent with, rather than direct proof of, herbivore–omnivore feeding behavior.

The strong correlations between intestine length, general intestinal ratio, and relative intestinal length further indicate coordinated digestive system scaling. Similar intestinal proportions have been documented in herbivorous tropical fishes, where elongated digestive tracts are associated with enhanced retention time and improved utilization of plant-derived material [16,36]. Consequently, the intestinal morphology observed in this study supports the capacity of *P. brachypomus* to efficiently exploit plant-based diets commonly used in semi-intensive aquaculture systems.

From a production perspective, these findings have important implications for feed formulation and nutritional management. Larger fish with proportionally elongated digestive systems may exhibit improved utilization of high-fibre ingredients and reduced dependence on expensive protein sources. This characteristic is particularly relevant for sustainable aquaculture development in tropical regions, where reducing fishmeal inclusion remains a major economic and environmental priority [37,38].

### 4.4. Implications for Commercial Processing and Harvest Optimization

The present study indicates that commercial weight influences processing performance in *P. brachypomus,* particularly regarding edible tissue recovery. The substantial increase in *EPT* and *EPC* with increasing body weight indicates that harvest size selection can markedly alter product recovery efficiency.

Fresh weight emerged as the dominant predictor of edible recovery in all regression models, explaining nearly 60% of *EPT* variability by itself. The negligible improvement obtained by adding additional morphometric predictors suggests that fresh weight effectively integrates overall somatic development, muscle deposition, and body conformation. Therefore, harvest weight constitutes the most practical and reliable indicator of processing efficiency under commercial conditions [39]. The supplementary polynomial analysis (Section 3.3; Supplementary Figure S1) further indicates that this relationship, while strongly positive overall, shows a statistically significant degree of curvature (quadratic term *p* = 0.0025), with the rate of *EPT* increase tending to moderate at the highest body weights evaluated (approximately 480–530 g). This nuance does not alter the principal conclusion that *FW* is the dominant predictor of *EPT*, but it suggests that gains in edible recovery per additional unit of body weight may not be perfectly constant across the entire commercial range.

The increasing divergence between carcass yield and edible recovery across categories highlights an important biological trade-off during growth. As fish become larger, greater visceral development reduces eviscerated yield, but simultaneous muscular expansion substantially enhances edible tissue recovery. Consequently, evaluating processing performance solely based on carcass yield may underestimate the processing value of larger specimens. Instead, edible recovery indicators provide a more realistic measure of processing yield in fillet-oriented production systems [40]. We reiterate that this is a processing-yield-based, not an economic-profitability, conclusion; estimating industrial profitability would require additional feed-cost and market-price data not collected in the present study.

The tighter confidence intervals observed at intermediate body weights suggest that fish within the C2 category may provide more predictable processing outcomes, whereas the C3 category maximizes edible recovery. Thus, the optimal harvest range will ultimately depend on market objectives. Producers targeting whole-fish commercialization may benefit from smaller categories with higher carcass yield, whereas processors focused on fillet production and value-added products may achieve a higher edible-tissue yield with larger fish, subject to the economic caveats noted above [29].

Overall, the results indicate that ontogenetic changes in body composition and morphometric allocation directly influence commercial processing performance in *P. brachypomus.* These findings provide a quantitative basis for optimizing harvest strategies, improving processing standardisation, and supporting evidence-based management decisions in tropical freshwater aquaculture systems.

A few factors limit how broadly these results apply. All specimens came from a single semi-intensive earthen-pond system under uniform management, so extrapolation to other watersheds, production systems, or culture methods should be made cautiously. Sex was not stratified, as the species lacks reliable external sexual characteristics at the sizes studied, and sampling was confined to a single harvest period rather than multiple production cycles. Although several variables departed from normality, non-parametric tests reproduced the same conclusions, confirming robustness. Finally, high multicollinearity among morphometric predictors limits interpretation of individual coefficients in the multi-predictor regression models.

## 5. Conclusions

The commercial weight categories in *Piaractus brachypomus* were associated with progressive changes in morphometric traits, intestinal development, and processing yield indicators. Most external morphometric variables increased significantly with body weight, particularly trunk length and fin dimensions, while tail length remained relatively stable across categories. Multivariate analyses indicated clear separation among commercial classes, suggesting that the evaluated categories correspond to distinguishable growth stages characterized by differences in body conformation and tissue distribution.

Intestinal morphometric analyses demonstrated increasing intestine length and intestinal ratios with growth, with relative intestinal length values above 2.0 in all categories. The observed allometric patterns suggest that digestive development changes during ontogeny and may be associated with the feeding ecology of the species, although confirmation of this specific association would require dietary analyses beyond the scope of the present study. Correlation analyses further revealed strong positive relationships among body size variables, intestinal traits, and edible recovery indicators, indicating coordinated development between somatic and digestive structures.

Processing yield analyses showed contrasting tendencies among evaluated indicators. Carcass yield decreased moderately with increasing body weight, whereas edible part recovery increased substantially in larger fish. Regression models indicated that fresh weight explained an important proportion of the variability in edible recovery, while additional morphometric predictors provided only limited improvements in model performance. A supplementary analysis indicated that the increase in edible recovery with body weight is not perfectly linear across the entire range and shows some moderation at the highest evaluated weights, although fresh weight remains by far the strongest single predictor.

These results suggest that, within semi-intensive earthen-pond *P. brachypomus* production in the Satipo Province of Junín, harvest weight constitutes a practical indicator for estimating processing performance under commercial conditions. Extrapolation of these findings to other Amazonian watersheds, sex-unstratified populations, or alternative culture systems should be validated independently.

## Figures and Tables

**Table 1:** Morphometric and digestive variables evaluated in *Piaractus brachypomus*.

Abbreviation	Variable	Definition
*FW*	Fresh weight	Total body mass of the fish prior to processing (g).
*TL*	Total length	Distance from the anterior tip of the snout to the posterior end of the caudal fin when fully extended.
*SL*	Standard length	Distance from the anterior tip of the snout to the posterior end of the vertebral column (base of caudal fin).
*TrL*	Trunk length	Distance from the posterior margin of the operculum to the anterior insertion of the anal fin.
*TaL*	Tail length	Distance from the anterior insertion of the anal fin to the base of the caudal fin, measured from the base of the caudal peduncle to the tip of the longest lobe when fully extended.
*DFL*	Dorsal fin length	Maximum extension length of the dorsal fin.
*PFL*	Pectoral fin length	Maximum extension length of the pectoral fin.
*PvFL*	Pelvic fin length	Maximum extension length of the pelvic fin.
*AFL*	Anal fin length	Maximum extension length of the anal fin.
*CFL*	Caudal fin length	Maximum extension length of the caudal fin.
*IL*	Intestinal length	Total length of the digestive tract from the pyloric sphincter to the anus after dissection.
*GR*	General intestinal ratio	Ratio between intestinal length and total length (*IL*/*TL*).
*RIL*	Relative intestinal length	Ratio between intestinal length and standard length (*IL*/*SL*).

**Table 2:** External morphometric characteristics (mean ± SD) of *Piaractus brachypomus* across three commercial weight categories. Different lowercase letters within rows indicate significant differences (Duncan test, *p* < 0.05). ns = not significant.

Variable	C1: 251–350 g	C2: 351–450 g	C3: 451–550 g	Sig.
Fresh Weight (g)	322.78 ± 15.46 ^a^	425.52 ± 20.09 ^b^	493.00 ± 23.23 ^c^	** (F = 466.22, *p* < 0.0001)
Total Length (cm)	23.82 ± 0.52 ^a^	25.58 ± 0.47 ^b^	27.31 ± 0.83 ^c^	** (F = 194.13, *p* < 0.0001)
Standard Length (cm)	19.08 ± 0.83 ^a^	20.42 ± 0.43 ^b^	22.21 ± 0.68 ^c^	** (F = 138.33, *p* < 0.0001)
Trunk Length (cm)	13.82 ± 0.43 ^a^	15.07 ± 0.47 ^b^	18.90 ± 1.36 ^c^	** (F = 232.58, *p* < 0.0001)
Tail Length (cm)	6.10 ± 0.50 ^a^	6.20 ± 0.54 ^a^	6.14 ± 0.36 ^a^	ns (F = 0.283, *p* = 0.7544)
Dorsal Fin (cm)	3.91 ± 0.19 ^a^	4.24 ± 0.25 ^b^	4.54 ± 0.40 ^c^	** (F = 28.00, *p* < 0.0001)
Pectoral Fin (cm)	3.60 ± 0.31 ^a^	4.14 ± 0.23 ^b^	4.54 ± 0.20 ^c^	** (F = 88.67, *p* < 0.0001)
Pelvic Fin (cm)	3.00 ± 0.00 ^a^	3.52 ± 0.18 ^b^	3.81 ± 0.38 ^c^	** (F = 73.44, *p* < 0.0001)
Anal Fin (cm)	5.91 ± 0.19 ^a^	6.74 ± 0.25 ^b^	7.20 ± 0.56 ^c^	** (F = 77.67, *p* < 0.0001)
Caudal Fin (cm)	10.04 ± 0.61 ^a^	11.16 ± 0.37 ^b^	11.74 ± 0.46 ^c^	** (F = 77.34, *p* < 0.0001)

** Indicates a highly significant overall effect of weight category based on one-way ANOVA (*p* < 0.0001). ns = not significant.

**Table 3:** Intestinal morphometric traits (mean ± SD) of *Piaractus brachypomus* across three commercial weight categories. Different lowercase letters indicate significant differences (Duncan test, *p* < 0.05).

Trait	C1: 251–350 g	C2: 351–450 g	C3: 451–550 g	Sig.
Intestine Length (cm)	38.83 ± 5.04 a	45.08 ± 3.30 b	54.80 ± 3.24 c	**
*IL*/*TL* Ratio (General)	1.63 ± 0.22 a	1.76 ± 0.12 b	2.01 ± 0.12 c	**
*IL*/*SL* Ratio (Relative *IL*)	2.04 ± 0.29 a	2.21 ± 0.15 b	2.47 ± 0.16 c	**

** Indicates a highly significant overall effect of commercial weight category based on one-way ANOVA (*p* < 0.0001).

**Table 4:** Processing yield indicators (mean ± SD) of *Piaractus brachypomus* across three commercial weight categories. Different lowercase letters indicate significant differences (Duncan test, *p* < 0.05). ns = not significant.

Yield Indicator	C1: 251–350 g	C2: 351–450 g	C3: 451–550 g	Sig.
Carcass Yield (%)	87.09 ± 6.38 a	84.77 ± 4.80 a	79.72 ± 6.79 b	** (F = 9.68, *p* = 0.0002)
Fried Carcass Yield (%)	59.79 ± 3.83 a	65.77 ± 2.12 b	66.10 ± 3.11 b	** (F = 32.91, *p* < 0.0001)
Edible Part/Total Weight (%)	37.05 ± 3.32 a	47.28 ± 2.17 b	49.54 ± 3.24 c	** (F = 126.66, *p* < 0.0001)
Edible Part/Carcass Weight (%)	42.71 ± 4.38 a	55.92 ± 3.53 b	62.48 ± 5.70 c	** (F = 118.70, *p* < 0.0001)

** Indicates a statistically significant overall effect of commercial weight category based on one-way ANOVA. Exact F and p values are reported in the last column. ns = not significant.

**Table 5:** Multiple linear regression models for carcass yield (*CY*) and edible part/total weight (*EPT*) in *Piaractus brachypomus* (*n* = 75). Predictors were added hierarchically. Models are compared using R^2^, adjusted R^2^, F-test, *p*-value, AIC, and VIF. Individual coefficients from multi-predictor models were not interpreted due to severe multicollinearity.

Model	Predictors	R^2^	Adj. R^2^	F-Value	*p* (F)	AIC	VIF Range
Carcass Yield (*CY*)							
M1	*FW*	0.292	0.282	30.12	<0.0001	450.8	—
M2	*FW* + *TrL*	0.329	0.310	17.65	<0.0001	449.2	104.1
M3	*FW* + *TrL* + *IL*	0.358	0.331	13.18	<0.0001	448.7	139–143
M4	*FW* + *TrL* + *IL* + *SL*	0.358	0.321	9.75	<0.0001	450.6	126–199
Edible Part/Total Weight (*EPT*)							
M1	*FW*	0.594	0.588	106.63	<0.0001	383.5	—
M2	*FW* + *TrL*	0.603	0.592	54.66	<0.0001	384.0	104.1
M3	*FW* + *TrL* + *IL*	0.603	0.586	35.95	<0.0001	385.9	139–143
M4	*FW* + *TrL* + *IL* + *SL*	0.604	0.581	26.67	<0.0001	387.8	126–199

## Data Availability

The dataset supporting the results of this article is openly available in the Figshare repository at https://doi.org/10.6084/m9.figshare.32337198.

## Supplementary Materials

The following supporting information can be downloaded at: https://www.mdpi.com/article/10.3390/ani16152335/s1, Figure S1. Relationship between fresh weight (*FW*) and (left) carcass yield (*CY*), (right) edible part/total weight (*EPT*), *n* = 75. Dashed grey lines mark the category limits (350 g, 450 g). Black line: linear fit. Dashed red line: 2nd-degree polynomial fit. Table S1. Shapiro-Wilk Normality Test and Levene’s Homogeneity of Variance Test Results. Table S2. Complete Test Statistics: One-Way ANOVA (Parametric) and Kruskal-Wallis (Non-Parametric). Table S3. Tukey HSD Post-Hoc Test Results (Parametric Robustness Check vs. Duncan’s Test). Table S4. Games-Howell Post-Hoc Test Results (Non-Parametric-Robust Pairwise Comparisons). Table S5. Principal Component Analysis (PCA) Variable Loadings.

## Abbreviations

The following abbreviations are used in this manuscript:

**Abbreviation**	**Meaning**
*AFL*	Anal fin length
AIC	Akaike Information Criterion
ANOVA	Analysis of variance
C1	Commercial weight category 1: 251–350 g
C2	Commercial weight category 2: 351–450 g
C3	Commercial weight category 3: 451–550 g
*CFL*	Caudal fin length
CRD	Completely randomized design
*CY*	Carcass yield
*DFL*	Dorsal fin length
*EP*	Edible portion weight
*EPC*	Edible part relative to carcass weight
*EPT*	Edible part relative to total weight
*EW*	Eviscerated weight
*FCY*	Fried carcass yield
*FW*	Fresh weight
*FWc*	Fried carcass weight
*GR*	General intestinal ratio
*IL*	Intestinal length
PCA	Principal component analysis
*PFL*	Pectoral fin length
*PvFL*	Pelvic fin length
*RIL*	Relative intestinal length
*SL*	Standard length
*TaL*	Tail length
*TL*	Total length
*TrL*	Trunk length
