# Supplementary material for: Carcass Yield and Morphometric Characteristics of Semi-Intensive Pond-Cultured Piaractus brachypomus (Paco) at Three Commercial Weight Ranges in the Central Jungle of Peru
Source: Animals (Basel). 2026 Jul 31;16(15):2335. doi: 10.3390/ani16152335 (PMC13463469; doi:10.3390/ani16152335)
Supplement: Supplementary file 1 [file animals-16-02335-s001.zip › animals-4360379-supplementary.pdf]

## SUPPLEMENTARY MATERIAL

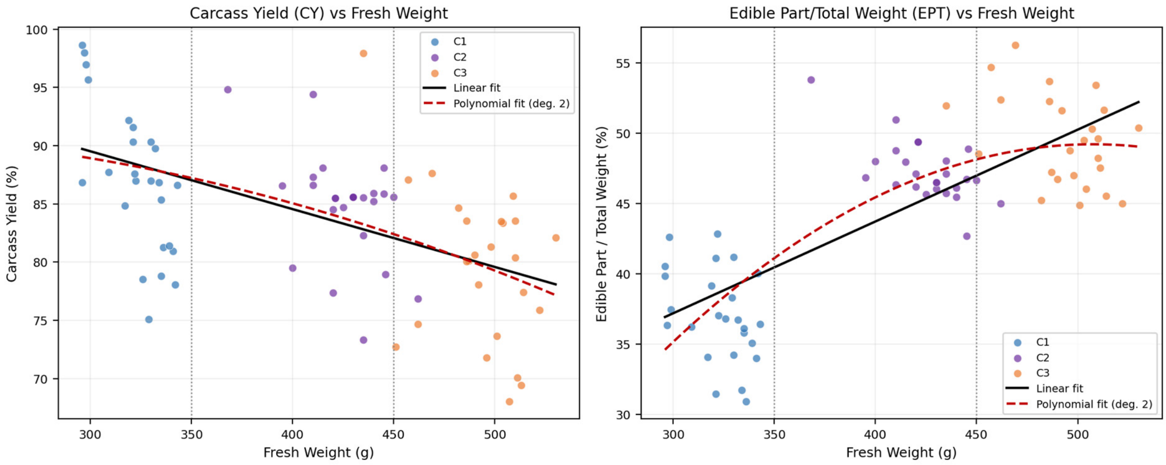


*Figure S1. Relationship between fresh weight (FW) and (left) carcass yield (CY), (right) edible part/total weight (EPT), n=75. Dashed grey lines mark the category limits (350 g, 450 g). Black line: linear fit. Dashed red line: 2nd-degree polynomial fit.*

*Table S1. Shapiro-Wilk Normality Test and Levene's Homogeneity of Variance Test Results*

| Variable | Description | Shapiro-Wilk W | Shapiro-Wilk p | Normal (p>0.05) | Levene F | Levene p |
|---|---|---|---|---|---|---|
| FW | Fresh Weight (g) | 0.9139 | 0.0001 | No | 1.0145 | 0.3677 |
| TL | Total Length (cm) | 0.967 | 0.0475 | No | 1.9037 | 0.1564 |
| SL | Standard Length (cm) | 0.9629 | 0.027 | No | 2.3776 | 0.1 |
| TrL | Trunk Length (cm) | 0.8621 | 0 | No | 24.3706 | 0 |
| TaL | Tail Length (cm) | 0.8866 | 0 | No | 1.3773 | 0.2588 |
| DFL | Dorsal Fin (cm) | 0.8368 | 0 | No | 7.0144 | 0.0016 |
| PFL | Pectoral Fin (cm) | 0.904 | 0 | No | 2.9585 | 0.0582 |
| PvFL | Pelvic Fin (cm) | 0.818 | 0 | No | 12.1322 | 0 |
| AFL | Anal Fin (cm) | 0.8795 | 0 | No | 2.0758 | 0.1329 |
| CFL | Caudal Fin (cm) | 0.8884 | 0 | No | 0.8869 | 0.4164 |
| IL | Intestine Length (cm) | 0.9767 | 0.1811 | Yes | 1.6755 | 0.1944 |
| GR | IL/TL Ratio (General) | 0.9797 | 0.2706 | Yes | 3.4006 | 0.0388 |
| RIL | IL/SL Ratio (Relative) | 0.9706 | 0.078 | Yes | 4.371 | 0.0162 |
| CY | Carcass Yield (%) | 0.9759 | 0.1634 | Yes | 2.061 | 0.1348 |
| FCY | Fried Carcass Yield (%) | 0.9609 | 0.0206 | No | 5.537 | 0.0058 |
| EPT | Edible Part/Total Weight (%) | 0.9475 | 0.0036 | No | 3.3717 | 0.0398 |
| EPC | Edible Part/Carcass Weight (%) | 0.9746 | 0.1358 | Yes | 2.7096 | 0.0734 |

*Table S2. Complete Test Statistics: One-Way ANOVA (Parametric) and Kruskal-Wallis (Non-Parametric)*

| Variable | Description | ANOVA F | df1,df2 | ANOVA p | ANOVA Sig. | Kruskal-Wallis H | KW df | KW p |
|---|---|---|---|---|---|---|---|---|
| FW | Fresh Weight (g) | 466.218 | 2, 72 | <0.0001 | Yes | 64.679 | 2 | <0.0001 |
| TL | Total Length (cm) | 194.132 | 2, 72 | <0.0001 | Yes | 64.861 | 2 | <0.0001 |
| SL | Standard Length (cm) | 138.333 | 2, 72 | <0.0001 | Yes | 59.545 | 2 | <0.0001 |
| TrL | Trunk Length (cm) | 232.58 | 2, 72 | <0.0001 | Yes | 65.505 | 2 | <0.0001 |
| TaL | Tail Length (cm) | 0.283 | 2, 72 | 0.7544 | No (ns) | 0.092 | 2 | 0.9548 |
| DFL | Dorsal Fin (cm) | 27.997 | 2, 72 | <0.0001 | Yes | 34.148 | 2 | <0.0001 |
| PFL | Pectoral Fin (cm) | 88.665 | 2, 72 | <0.0001 | Yes | 54.178 | 2 | <0.0001 |
| PvFL | Pelvic Fin (cm) | 73.438 | 2, 72 | <0.0001 | Yes | 59.329 | 2 | <0.0001 |

| | | | | | | | | |
|---|---|---|---|---|---|---|---|---|
| AFL | Anal Fin (cm) | 77.671 | 2, 72 | <0.0001 | Yes | 51.769 | 2 | <0.0001 |
| CFL | Caudal Fin (cm) | 77.344 | 2, 72 | <0.0001 | Yes | 51.128 | 2 | <0.0001 |
| IL | Intestine Length (cm) | 103.767 | 2, 72 | <0.0001 | Yes | 56.59 | 2 | <0.0001 |
| GR | IL/TL Ratio (General) | 36.538 | 2, 72 | <0.0001 | Yes | 39.283 | 2 | <0.0001 |
| RIL | IL/SL Ratio (Relative) | 26.47 | 2, 72 | <0.0001 | Yes | 32.486 | 2 | <0.0001 |
| CY | Carcass Yield (%) | 9.678 | 2, 72 | 0.0002 | Yes | 16.654 | 2 | 0.0002 |
| FCY | Fried Carcass Yield (%) | 32.906 | 2, 72 | <0.0001 | Yes | 32.601 | 2 | <0.0001 |
| EPT | Edible Part/Total Weight (%) | 126.657 | 2, 72 | <0.0001 | Yes | 51.882 | 2 | <0.0001 |
| EPC | Edible Part/Carcass Weight (%) | 118.701 | 2, 72 | <0.0001 | Yes | 56.659 | 2 | <0.0001 |

*Table S3. Tukey HSD Post-Hoc Test Results (Parametric Robustness Check vs. Duncan's Test)*

| Variable | Group 1 | Group 2 | Mean Difference | Adj. p-value | 95% CI Lower | 95% CI Upper |
|---|---|---|---|---|---|---|
| FW | C1 | C2 | 102.744 | 0 | 89.308 | 116.18 |
| FW | C1 | C3 | 170.224 | 0 | 156.788 | 183.66 |
| FW | C2 | C3 | 67.48 | 0 | 54.044 | 80.916 |
| TL | C1 | C2 | 1.761 | 0 | 1.337 | 2.185 |
| TL | C1 | C3 | 3.493 | 0 | 3.069 | 3.917 |
| TL | C2 | C3 | 1.732 | 0 | 1.308 | 2.156 |
| SL | C1 | C2 | 1.344 | 0 | 0.892 | 1.796 |
| SL | C1 | C3 | 3.132 | 0 | 2.68 | 3.584 |
| SL | C2 | C3 | 1.788 | 0 | 1.336 | 2.24 |
| TrL | C1 | C2 | 1.252 | 0 | 0.665 | 1.839 |
| TrL | C1 | C3 | 5.08 | 0 | 4.493 | 5.667 |
| TrL | C2 | C3 | 3.828 | 0 | 3.241 | 4.415 |
| TaL | C1 | C2 | 0.1 | 0.7363 | -0.22 | 0.42 |
| TaL | C1 | C3 | 0.04 | 0.952 | -0.28 | 0.36 |
| TaL | C2 | C3 | -0.06 | 0.8953 | -0.38 | 0.26 |
| DFL | C1 | C2 | 0.328 | 0.0006 | 0.128 | 0.528 |
| DFL | C1 | C3 | 0.624 | 0 | 0.424 | 0.824 |
| DFL | C2 | C3 | 0.296 | 0.002 | 0.096 | 0.496 |
| PFL | C1 | C2 | 0.544 | 0 | 0.374 | 0.714 |
| PFL | C1 | C3 | 0.94 | 0 | 0.77 | 1.11 |
| PFL | C2 | C3 | 0.396 | 0 | 0.226 | 0.566 |
| PvFL | C1 | C2 | 0.52 | 0 | 0.358 | 0.682 |
| PvFL | C1 | C3 | 0.812 | 0 | 0.65 | 0.974 |
| PvFL | C2 | C3 | 0.292 | 0.0002 | 0.13 | 0.454 |
| AFL | C1 | C2 | 0.832 | 0 | 0.581 | 1.083 |
| AFL | C1 | C3 | 1.292 | 0 | 1.04 | 1.544 |
| AFL | C2 | C3 | 0.46 | 0.0001 | 0.208 | 0.712 |
| CFL | C1 | C2 | 1.12 | 0 | 0.787 | 1.453 |
| CFL | C1 | C3 | 1.7 | 0 | 1.367 | 2.033 |
| CFL | C2 | C3 | 0.58 | 0.0002 | 0.247 | 0.913 |
| IL | C1 | C2 | 6.248 | 0 | 3.575 | 8.921 |
| IL | C1 | C3 | 15.968 | 0 | 13.295 | 18.641 |
| IL | C2 | C3 | 9.72 | 0 | 7.047 | 12.393 |
| GR | C1 | C2 | 0.13 | 0.0137 | 0.023 | 0.237 |
| GR | C1 | C3 | 0.378 | 0 | 0.27 | 0.485 |
| GR | C2 | C3 | 0.248 | 0 | 0.14 | 0.355 |
| RIL | C1 | C2 | 0.168 | 0.0166 | 0.026 | 0.31 |
| RIL | C1 | C3 | 0.429 | 0 | 0.287 | 0.572 |
| RIL | C2 | C3 | 0.261 | 0.0001 | 0.119 | 0.404 |
| CY | C1 | C2 | -2.32 | 0.3698 | -6.417 | 1.777 |
| CY | C1 | C3 | -7.365 | 0.0002 | -11.462 | -3.268 |

| | | | | | | |
|---|---|---|---|---|---|---|
| CY | C2 | C3 | -5.045 | 0.0119 | -9.142 | -0.948 |
| FCY | C1 | C2 | 5.981 | 0 | 3.885 | 8.077 |
| FCY | C1 | C3 | 6.312 | 0 | 4.216 | 8.408 |
| FCY | C2 | C3 | 0.331 | 0.9245 | -1.765 | 2.427 |
| EPT | C1 | C2 | 10.229 | 0 | 8.227 | 12.23 |
| EPT | C1 | C3 | 12.49 | 0 | 10.488 | 14.491 |
| EPT | C2 | C3 | 2.261 | 0.023 | 0.26 | 4.262 |
| EPC | C1 | C2 | 13.21 | 0 | 10.082 | 16.338 |
| EPC | C1 | C3 | 19.77 | 0 | 16.642 | 22.898 |
| EPC | C2 | C3 | 6.561 | 0 | 3.433 | 9.689 |

*Table S4. Games-Howell Post-Hoc Test Results (Non-Parametric-Robust Pairwise Comparisons)*

| Variable | Group A | Group B | Mean Diff (A-B) | SE | T | df (Welch) | p-value | Significant | Hedges g |
|---|---|---|---|---|---|---|---|---|---|
| FW | C1 | C2 | -102.744 | 5.0692 | -20.268 | 45.04 | <0.0001 | Yes | -5.643 |
| FW | C1 | C3 | -170.224 | 5.5803 | -30.504 | 41.77 | <0.0001 | Yes | -8.492 |
| FW | C2 | C3 | -67.48 | 6.142 | -10.987 | 47.02 | <0.0001 | Yes | -3.059 |
| TL | C1 | C2 | -1.761 | 0.14 | -12.581 | 47.6 | <0.0001 | Yes | -3.502 |
| TL | C1 | C3 | -3.493 | 0.1955 | -17.863 | 40.19 | <0.0001 | Yes | -4.973 |
| TL | C2 | C3 | -1.732 | 0.1909 | -9.073 | 38.04 | <0.0001 | Yes | -2.526 |
| SL | C1 | C2 | -1.344 | 0.187 | -7.186 | 35.72 | <0.0001 | Yes | -2.001 |
| SL | C1 | C3 | -3.132 | 0.2152 | -14.555 | 46.19 | <0.0001 | Yes | -4.052 |
| SL | C2 | C3 | -1.788 | 0.1606 | -11.132 | 40.24 | <0.0001 | Yes | -3.099 |
| TrL | C1 | C2 | -1.252 | 0.1268 | -9.872 | 47.7 | <0.0001 | Yes | -2.748 |
| TrL | C1 | C3 | -5.08 | 0.2858 | -17.776 | 28.74 | <0.0001 | Yes | -4.949 |
| TrL | C2 | C3 | -3.828 | 0.288 | -13.291 | 29.54 | <0.0001 | Yes | -3.7 |
| TaL | C1 | C2 | -0.1 | 0.1474 | -0.678 | 47.74 | 0.7772 | No | -0.189 |
| TaL | C1 | C3 | -0.04 | 0.1233 | -0.324 | 43.43 | 0.9437 | No | -0.09 |
| TaL | C2 | C3 | 0.06 | 0.1296 | 0.463 | 41.69 | 0.8889 | No | 0.129 |
| DFL | C1 | C2 | -0.328 | 0.0633 | -5.179 | 44.12 | <0.0001 | Yes | -1.442 |
| DFL | C1 | C3 | -0.624 | 0.0885 | -7.047 | 34.05 | <0.0001 | Yes | -1.962 |
| DFL | C2 | C3 | -0.296 | 0.095 | -3.115 | 40.68 | 0.0092 | Yes | -0.867 |
| PFL | C1 | C2 | -0.544 | 0.077 | -7.061 | 44.76 | <0.0001 | Yes | -1.966 |
| PFL | C1 | C3 | -0.94 | 0.0733 | -12.831 | 41.27 | <0.0001 | Yes | -3.572 |
| PFL | C2 | C3 | -0.396 | 0.0614 | -6.45 | 46.93 | <0.0001 | Yes | -1.796 |
| PvFL | C1 | C2 | -0.52 | 0.0351 | -14.807 | 24 | <0.0001 | Yes | -4.122 |
| PvFL | C1 | C3 | -0.812 | 0.0753 | -10.777 | 24 | <0.0001 | Yes | -3 |
| PvFL | C2 | C3 | -0.292 | 0.0831 | -3.513 | 33.96 | 0.0036 | Yes | -0.978 |
| AFL | C1 | C2 | -0.832 | 0.0637 | -13.051 | 44.52 | <0.0001 | Yes | -3.633 |
| AFL | C1 | C3 | -1.292 | 0.1182 | -10.933 | 29.55 | <0.0001 | Yes | -3.044 |
| AFL | C2 | C3 | -0.46 | 0.1229 | -3.743 | 33.57 | 0.0019 | Yes | -1.042 |
| CFL | C1 | C2 | -1.12 | 0.1433 | -7.816 | 39.78 | <0.0001 | Yes | -2.176 |
| CFL | C1 | C3 | -1.7 | 0.1529 | -11.121 | 44.55 | <0.0001 | Yes | -3.096 |
| CFL | C2 | C3 | -0.58 | 0.1185 | -4.896 | 46.12 | <0.0001 | Yes | -1.363 |
| IL | C1 | C2 | -6.248 | 1.205 | -5.185 | 41.41 | <0.0001 | Yes | -1.444 |
| IL | C1 | C3 | -15.968 | 1.1981 | -13.327 | 40.95 | <0.0001 | Yes | -3.71 |
| IL | C2 | C3 | -9.72 | 0.9254 | -10.503 | 47.98 | <0.0001 | Yes | -2.924 |
| GR | C1 | C2 | -0.13 | 0.0494 | -2.632 | 37.22 | 0.0322 | Yes | -0.733 |
| GR | C1 | C3 | -0.378 | 0.0496 | -7.616 | 37.59 | <0.0001 | Yes | -2.12 |
| GR | C2 | C3 | -0.248 | 0.0338 | -7.316 | 47.99 | <0.0001 | Yes | -2.037 |
| RIL | C1 | C2 | -0.168 | 0.0652 | -2.575 | 36.85 | 0.0369 | Yes | -0.717 |
| RIL | C1 | C3 | -0.429 | 0.0659 | -6.512 | 37.83 | <0.0001 | Yes | -1.813 |
| RIL | C2 | C3 | -0.261 | 0.0448 | -5.836 | 47.91 | <0.0001 | Yes | -1.625 |
| CY | C1 | C2 | 2.32 | 1.5969 | 1.453 | 44.56 | 0.3232 | No | 0.404 |
| CY | C1 | C3 | 7.365 | 1.8642 | 3.951 | 47.82 | 0.0007 | Yes | 1.1 |
| CY | C2 | C3 | 5.045 | 1.6632 | 3.033 | 43.17 | 0.0112 | Yes | 0.844 |
| FCY | C1 | C2 | -5.981 | 0.8744 | -6.84 | 37.45 | <0.0001 | Yes | -1.904 |
| FCY | C1 | C3 | -6.312 | 0.9854 | -6.405 | 46.06 | <0.0001 | Yes | -1.783 |

| | | | | | | | | | |
|---|---|---|---|---|---|---|---|---|---|
| FCY | C2 | C3 | -0.331 | 0.7518 | -0.44 | 42.35 | 0.899 | No | -0.122 |
| EPT | C1 | C2 | -10.229 | 0.7932 | -12.895 | 41.35 | <0.0001 | Yes | -3.59 |
| EPT | C1 | C3 | -12.49 | 0.9277 | -13.463 | 47.97 | <0.0001 | Yes | -3.748 |
| EPT | C2 | C3 | -2.261 | 0.7799 | -2.899 | 41.93 | 0.016 | Yes | -0.807 |
| EPC | C1 | C2 | -13.21 | 1.1245 | -11.747 | 45.95 | <0.0001 | Yes | -3.27 |
| EPC | C1 | C3 | -19.77 | 1.4367 | -13.761 | 45.01 | <0.0001 | Yes | -3.831 |
| EPC | C2 | C3 | -6.561 | 1.3405 | -4.894 | 40.06 | <0.0001 | Yes | -1.363 |

*Table S5. Principal Component Analysis (PCA) Variable Loadings*

| Variable | Description | PC1 Loading | PC2 Loading |
|---|---|---|---|
| FW | Fresh Weight (g) | 0.974 | 0.046 |
| TL | Total Length (cm) | 0.949 | 0.062 |
| SL | Standard Length (cm) | 0.921 | 0.133 |
| TrL | Trunk Length (cm) | 0.897 | -0.143 |
| TaL | Tail Length (cm) | 0.1 | 0.984 |
| DFL | Dorsal Fin (cm) | 0.753 | -0.215 |
| PFL | Pectoral Fin (cm) | 0.904 | 0.051 |
| PvFL | Pelvic Fin (cm) | 0.859 | -0.103 |
| AFL | Anal Fin (cm) | 0.859 | 0.027 |
| CFL | Caudal Fin (cm) | 0.86 | -0.017 |
